# The Exosomal lncRNA KLF3-AS1 From Ischemic Cardiomyocytes Mediates IGF-1 Secretion by MSCs to Rescue Myocardial Ischemia-Reperfusion Injury

**DOI:** 10.3389/fcvm.2021.671610

**Published:** 2021-09-21

**Authors:** Gecai Chen, Aihuan Yue, Meixiang Wang, Zhongbao Ruan, Li Zhu

**Affiliations:** ^1^Department of Cardiology, Taizhou People's Hospital, Taizhou, China; ^2^Taizhou Mabtech Pharmaceutical Co., Ltd., Taizhou, China

**Keywords:** KLF3-AS1, exosome, mesenchymal stem cell, IGF-1, myocardial ischemia-reperfusion injury

## Abstract

The purpose of the study was to explore the mechanism by which myocardial ischemia-reperfusion (I/R) injury-induced exosomes modulate mesenchymal stem cells (MSCs) to regulate myocardial injury. In this study, we established an I/R injury model *in vivo* and a hypoxia-reoxygenation (H/R) model *in vitro*. Then, exosomes isolated from H/R-exposed H9c2 cells were characterized using transmission electron microscopy (TEM), nanoparticle tracking analysis (NTA), and Western blot analysis. CCK-8 assays and flow cytometry were performed to assess cell injury. ELISA was applied to determine the level of insulin-like growth factor 1 (IGF-1). Echocardiography was used to assess cardiac function *in vivo*. HE staining and TUNEL assays were conducted to analyze myocardial injury *in vivo*. In the present study, H/R-exposed H9c2 cells induced IGF-1 secretion from MSCs to inhibit cell myocardial injury. Moreover, exosomes derived from H/R-exposed H9c2 cells were introduced to MSCs to increase IGF-1 levels. The lncRNA KLF3-AS1 was dramatically upregulated in exosomes derived from H/R-treated H9c2 cells. Functional experiments showed that the exosomal lncRNA KLF3-AS1 promoted IGF-1 secretion from MSCs and increased H9c2 cell viability. In addition, miR-23c contains potential binding sites for both KLF3-AS1 and STAT5B, and miR-23c directly bound to the 3'-UTRs of KLF3-AS1 and STAT5B. Furthermore, the lncRNA KLF3-AS1 promoted IGF-1 secretion from MSCs and rescued myocardial cell injury *in vivo* and *in vitro* by upregulating STAT5B expression. The lncRNA KLF3-AS1 may serve as a new direction for the treatment of myocardial I/R injury.

## Introduction

Acute myocardial infarction is considered as one of the leading causes of death worldwide due to its high morbidity and mortality rates (1). Substantial advances have been achieved in the clinical treatment of acute myocardial infarction, such as cutaneous coronary intervention, coronary artery bypass grafting, and drug therapy (2, 3). However, timely reperfusion induces myocardial ischemia-reperfusion (I/R) injury, which reduces the beneficial effect of reperfusion therapy (4). Therefore, it is necessary to explore the pathogenesis of apoptosis in myocardial I/R injury.

As a new therapeutic approach, mesenchymal stem cells (MSCs) have received increasing attention in the medical field (5, 6). MSCs are adult stem cells with the potential for self-renewal and multidifferentiation (7). MSCs present in various tissues, including bone marrow, adipose tissue, placental tissue, and umbilical cord tissue (7). Bone marrow-derived MSCs have been widely applied in animal and clinical studies (8, 9). Bone marrow-derived MSCs have been proven to participate in the processes of immune regulation and tissue injury healing. An extensive body of evidence shows that MSCs secrete many growth factors to stimulate the functions of host cells and then promote natural progenitor cell differentiation and the recovery of damaged cells (10, 11). However, *in vitro* culture of MSCs changes the signaling response of MSCs, which substantially reduces the ability of self-repair of damaged tissues (12). The use of inducers to recruit endogenous MSCs to damaged tissues has become a new method in regenerative medicine (13). For instance, chemokines induce MSCs to migrate to damaged tissue sites, form new tissues, or secrete cytokines to promote the self-regeneration of damaged tissue sites, thus achieving the repair effect (14). Insulin-like growth factor 1 (IGF-1) has a vital function in maintaining the structure and function of the body (15, 16). Recently, IGF-1 was revealed to be closely associated with the development and growth of cardiomyocytes by promoting cell growth and resisting cell death (17). Therefore, the secretion of IGF-1 by MSCs may be an effective strategy to retard myocardial I/R injury.

In recent years, exosomes have attracted the interest of numerous researchers worldwide. Emerging evidence confirms that exosomes have become a key regulator of angiogenesis and heart repair (18, 19). Exosomes are defined as vesicle-like bodies with a size of 30–200 nm that actively transport various biological signaling molecules, including transcription factors, long non-coding RNAs (lncRNAs), microRNAs (miRNAs), and messenger RNAs (mRNAs), into recipient cells (20). Exosomes are one of the important cell-cell signal communication pathways in the cardiovascular system. Exosomes produced in response to cardiac ischemic preconditioning exert a protective effect on the myocardium after I/R injury (21). LncRNAs are non-coding RNAs with a length of more than 200 nucleotides (22). LncRNAs are enriched and stable in exosomes and can be transferred into the recipient cells (23). The tissue specificity and developmental stage specificity of lncRNAs have been documented (24). The lncRNA rp11-617D20.1 is located on the antisense strand of the transcription initiation region of Krüppel-like factor 3 and is named lncRNA KLF3-AS1 (25). The lncRNA KLF3-AS1 has been reported to promote cartilage repair and inhibits chondrocyte apoptosis (26, 27). We observed an increased expression of the lncRNA KLF3-AS1 in ischemic myocardium-derived exosomes. However, researchers have not reported whether the lncRNA KLF3-AS1 is involved in MSC-induced IGF-1 secretion.

Therefore, the main objective of our research was to evaluate the exact functions and mechanisms of ischemic myocardium-derived exosomal lncRNA KLF3-AS1 in IGF-1 secretion by MSCs. We discovered that the exosomal lncRNA KLF3-AS1 induced IGF-1 secretion from MSCs through the miR-23c/STAT5B axis to inhibit myocardial injury.

## Materials and Methods

### Animals

The study was approved by the Institutional Animal Care and Use Committee of Taizhou People's Hospital. Sprague-Dawley (SD) rats were purchased from Jiangsu Animal Laboratory (Jiangsu, China). SD rats had free access to a standard diet and water at room temperature (18–25°C).

### Cell Culture

Four-week-old SD rats were euthanized by injecting 200 mg/kg of pentobarbital (Sigma, USA), and bone marrow MSCs were isolated from femurs and tibias in a sterile environment using a previously published method (28). Briefly, the femoral epiphyses were cut to expose the medullary cavity. Then, the medulla was rinsed with low-glucose Dulbecco's modified Eagle's medium (L-DMEM, Gibco, USA) containing 10% fetal bovine serum (FBS, Gibco, USA) and 1% penicillin-streptomycin (Sigma, USA) using a 118-gauge needle. The cells were gently passed through the needle 2–3 times to generate a single-cell suspension. The cell suspension was maintained in DMEM supplemented with 10% FBS and 1% penicillin-streptomycin under humidified conditions with 5% CO_2_ at 37°C. The adherent cells were changed medium every 3 days, and were passaged at a confluence of 90%. The cells at passage 4 were mainly uniform in morphology and spindle-shaped in appearance, which were the typical morphologies of bone marrow MSCs.

The rat myocardial cell line H9c2 was supplied by American Tissue Culture Collection (ATCC, USA). H9c2 cells were maintained in DMEM supplemented with 10% FBS and 1% penicillin-streptomycin at 37°C in 5% CO_2_.

### Cell Transfection and Treatment

For KLF3-AS1 overexpression, the pcDNA3.1 vector was used to construct the KLF3-AS1 plasmid by amplifying the coding sequence. KLF3-AS1 knockdown, miR-23c mimic, miR-23c inhibitor, and corresponding negative control (NC) were synthesized by GenePharma (Shanghai, China). Cells were transfected using Lipofectamine 2000 (Invitrogen, USA) according to the recommendations of the manufacturer.

H9c2 cells were maintained in DMEM deprived of glucose and serum in an anaerobic chamber with 1% O_2_, 94% N_2_, and 5% CO_2_ for 4 h. Then, the cells were maintained in DMEM supplemented with 10% FBS in a normal chamber with 95% O_2_ and 5% CO_2_ to establish the cellular hypoxia-reoxygenation (H/R) model *in vitro*. A Transwell (Corning, USA, 3412, 0.4 μm) coculture system was used to coculture H9c2 and MSC cells. H9c2 cells were seeded in 6-well plates at a density of 1 × 10^6^ cells per well. Then, MSCs were seeded in polycarbonate Transwell inserts in 6-well plates. H9c2 and MSC cells were cocultured for 48 h at 37°C with 5% CO_2_. H9c2 cells were treated with an anti-IGF-1 neutralizing antibody (IGF-1 Ab, R&D Systems, USA) and 100 ng/mL recombinant mouse IGF-1 (R&D Systems, USA) to assess the role of IGF-1 in H9c2 cells.

### Cell Counting Kit-8 Assay

Cell viability was analyzed using CCK-8 reagents (Beyotime, China). Cells were maintained in 96-well plates at a density of 1 × 10^4^ cells per well and exposed to the corresponding treatment. Afterward, the cells were incubated with the CCK-8 solution for 2 h. A microplate reader (Bio-Rad, USA) was used to measure the absorbance at 450 nm.

### Cell Apoptosis Assay

The cell apoptosis rate was examined using an Annexin V-FITC/PI apoptosis detection kit (Beyotime, China). Cells were seeded at a density of 2 × 10^5^/well, and the cells and culture medium were collected after different treatments. The collected cells were incubated with Annexin V-FITC and propidium iodide for 15 min in the dark. Finally, the rate of apoptosis was detected using a flow cytometer (BD Biosciences, USA).

### ELISA

The supernatant from cultured MSCs was collected, and the levels of secreted IGF-1 were quantified with an IGF-1 ELISA Kit (Abcam, USA).

### Exosome Isolation and Characterization

H9c2 cells were maintained in DMEM supplemented with exosome-depleted FBS for 48 h. A Hieff^TM^ Quick exosome isolation kit (YEASEN, China) was used to isolate the exosomes. Transmission electron microscopy (TEM) was used to observe the morphology and size of the exosomes, and nanoparticle tracking analysis (NTA) was performed to identify the particle size and diameter distribution.

### Exosome Labeling Assay

The extracted exosomes were labeled with a PKH-67 green fluorescent labeling kit (Sigma, USA). Briefly, exosomes were washed with a serum-free medium and resuspended in Diluent C to label them with the PKH-67 dye. Then, MSCs were cocultured with PKH-67-labeled exosomes for 48 h. DAPI (Invitrogen, USA) was added to stain cell nuclei. Exosome uptake was observed using a fluorescence microscope (Nikon, Japan).

### Western Blot

Total proteins from cells, exosomes, or tissues from the left ventricle were extracted by lysis in the RIPA lysis buffer (Beyotime, China), and protein concentrations were detected using a BCA kit (Beyotime, China). Equal amounts of protein samples were separated on SDS-PAGE gels and transferred to PVDF membranes. After blocking with 5% skim milk, membranes were incubated with the primary antibody. The following antibodies were used in the present study and purchased from Abcam: CD63 (ab217345, 1:1,000), CD81 (ab109201), STAT5B (ab178941, 1:1,000), and GAPDH (ab8245, 1:1,000). After an overnight incubation, membranes were probed with the secondary antibody at room temperature for 2 h. The protein signal was detected using ECL detection reagents (Thermo Scientific, CA, USA).

### RT-qPCR

Total RNA was extracted from cells, exosomes, or tissues with the TRIzol reagent (Invitrogen, USA), and cDNAs were synthesized using a Reverse Transcription Reagent Kit (Invitrogen, USA) according to the instructions of the manufacturer. Then, RT-qPCR was implemented using an ABI 7500-fast RT PCR system (Applied Biosystems) with a SYBR qPCR Mix Kit (Takara, China). Relative expression of KLF3-AS1 or mRNAs was evaluated using the 2^−ΔΔCt^ method and normalized to GAPDH. The primers used for RT-qPCR are as follows: IGF1 forward, 5′-GGCATTGTGGATGAGTGTTG-3′, IGF1 reverse, 5′-GCTGGGACTTCTGAGTCTTGG-3′; KLF3-AS1 forward, 5′-CTGTAGGCGCGCTCTTTCTTT-3′, KLF3-AS1 reverse, 5′-TCCGACCAAAGTTTGCCAAG-3′; STAT5B forward, 5′-AGCAGGCTTTTGGCATCAT-3′, STAT5B reverse, 5′-CCGTGTAGGCGAACTCAATTAG-3′; and GAPDH forward, 5′-AGAAGGCTGGGGCTCATTTG-3′, GAPDH reverse, 5′-AGGGGCCATCCACAGTCTTC-3′.

### Luciferase Reporter Assay

The wild-type (WT) and mutant (MUT) KLF3-AS1 or STAT5B fragments containing the potential binding sites for miR-23c were inserted into the luciferase reporter vector pMIR-GLO (Promega, USA). Cells were cotransfected with the reporter plasmids and miR-23c mimic or miR-23c inhibitor for 48 h. Subsequently, luciferase activity was detected with the Dual-Luciferase Reporter Assay (Promega, USA).

### RNA Immunoprecipitation Assay

RIP assays were performed using an Imprint RNA Immunoprecipitation Kit (Millipore, USA). The cells were lysed in a complete RNA lysis buffer and incubated with an Ago2 antibody or IgG overnight. Then, proteinase K was added to remove proteins and incubated at 55°C for 30 min, and immunoprecipitated RNA was assessed using RT-qPCR.

### Animal Experiments

Four-week-old SD rats were used to establish myocardial I/R models *in vivo*. Briefly, rats were anesthetized with pentobarbital (Sigma, USA), and the limbs and teeth were fixed. The trachea was intubated and connected to the artificial respirator. Next, ophthalmic scissors were used to make a small incision in the femoral vein, and a punctured tube containing heparin anticoagulant was carefully inserted. In the fourth intercostal space thoracotomy, a 7-0 silk thread was placed around the anterior descending branch of the left coronary artery. After 45 min of ischemia, the blood flow of the anterior descending branch was restored. SD rats were randomly divided into eight groups: Control group, I/R group, I/R+MSC group, I/R+MSC-MC(Control)-exo group, I/R+MSC-MC(H/R)-exo group, I/R+MSC-MC(KLF3-AS1 OE)-exo group, I/R+MSC-MC(H/R+KLF3-AS1 KD)-exo group, and I/R+MSC-MC(H/R)-exo+STAT5B-KD group. For the I/R+MSC group, MSCs were injected into the myocardium of the I/R models. For the I/R+MSC-MC(Control)-exo group, MSCs were treated with exosomes derived from myocardial cells H9c2, and then injected into the myocardium of the I/R models. For the, I/R+MSC-MC(H/R)-exo group, MSCs were treated with exosomes derived from H/R-induced myocardial cells H9c2, and then injected into the myocardium of the I/R models. For the I/R+MSC-MC(KLF3-AS1 OE)-exo group, MSCs were treated with exosomes derived from KLF3-AS1 overexpression plasmid-treated myocardial cells H9c2, and then injected into the myocardium of the I/R models. For the I/R+MSC-MC(H/R+KLF3-AS1 KD)-exo group, MSCs were treated with exosomes derived from H/R and KLF3-AS1 knockdown plasmid-treated myocardial cells H9c2, and then injected into the myocardium of the I/R models. For the I/R+MSC-MC(H/R)-exo+STAT5B-KD group, MSCs were treated with exosomes derived from H/R-induced myocardial cells H9c2, and transfected with STAT5B knockdown lentivirus, and then injected into the myocardium of the I/R models. After euthanasia, myocardial tissues were harvested and stored at −80°C until subsequent analysis.

### Echocardiographic Assessment

Rats were anesthetized with 3% isoflurane and fixed in the supine position on a thermostatic heating table. The limbs were connected to electrocardiogram electrodes and measured using the VEVO770 Ultrasound System (Visual Sonics Inc., Canada). The following variables were detected and averaged during three consecutive cardiac cycles: left ventricular ejection fraction (LVEF), left ventricular fractional shortening (LVFS), left ventricular systolic pressure (LVSP), and left ventricular end-diastolic pressure (LVEDP).

### 2,3,5-Triphenyltetrazole (TTC) Staining

After euthanasia, the heart tissues were rapidly excised and frozen at −20°C for 30 min. Then, the frozen left ventricles were cut into coronal sections at a thickness of 1 mm and dyed with a 1% TTC solution for 30 min. The slices were photographed, and the infarct sizes were analyzed using ImageJ software.

### Hematoxylin and Eosin Staining

Myocardial tissues were fixed with 4% paraffin, embedded, and sectioned. The slices were immersed in 0.5% hematoxylin for 5 min and stained with an eosin solution. Images were captured under a light microscope (Nikon, Japan).

### TUNEL Assay

Apoptosis of the myocardium was assessed using a Colorimetric TUNEL Apoptosis Assay Kit (Beyotime, China). Briefly, the tissue sections were permeabilized with DNase-free protease K for 15–30 min and treated with 3% H_2_O_2_ in PBS for 20 min_._ Subsequently, the sections were incubated with the TUNEL solution for 1 h and with streptavidin-H/RP for 30 min. Finally, the sections were incubated with a DAB solution and 3% hydrogen peroxide for 5–10 min. Images were captured under a light microscope (Nikon, Japan).

### Data Analysis

Experiments were repeated at least three times and the results are presented as the means ± standard deviations (SD). Statistical analyses were performed using GraphPad version 6.0 (GraphPad Software, Inc., USA). Comparisons between two groups were performed with the Student's *t*-test. The differences among multiple groups were analyzed using one-way ANOVA followed by a *post-hoc* test. *P* < 0.05 indicated a statistically significant difference.

## Results

### H/R-Exposed Myocardial Cells Induce IGF-1 Secretion From MSCs to Inhibit Myocardial Injury

**Figure 1:**
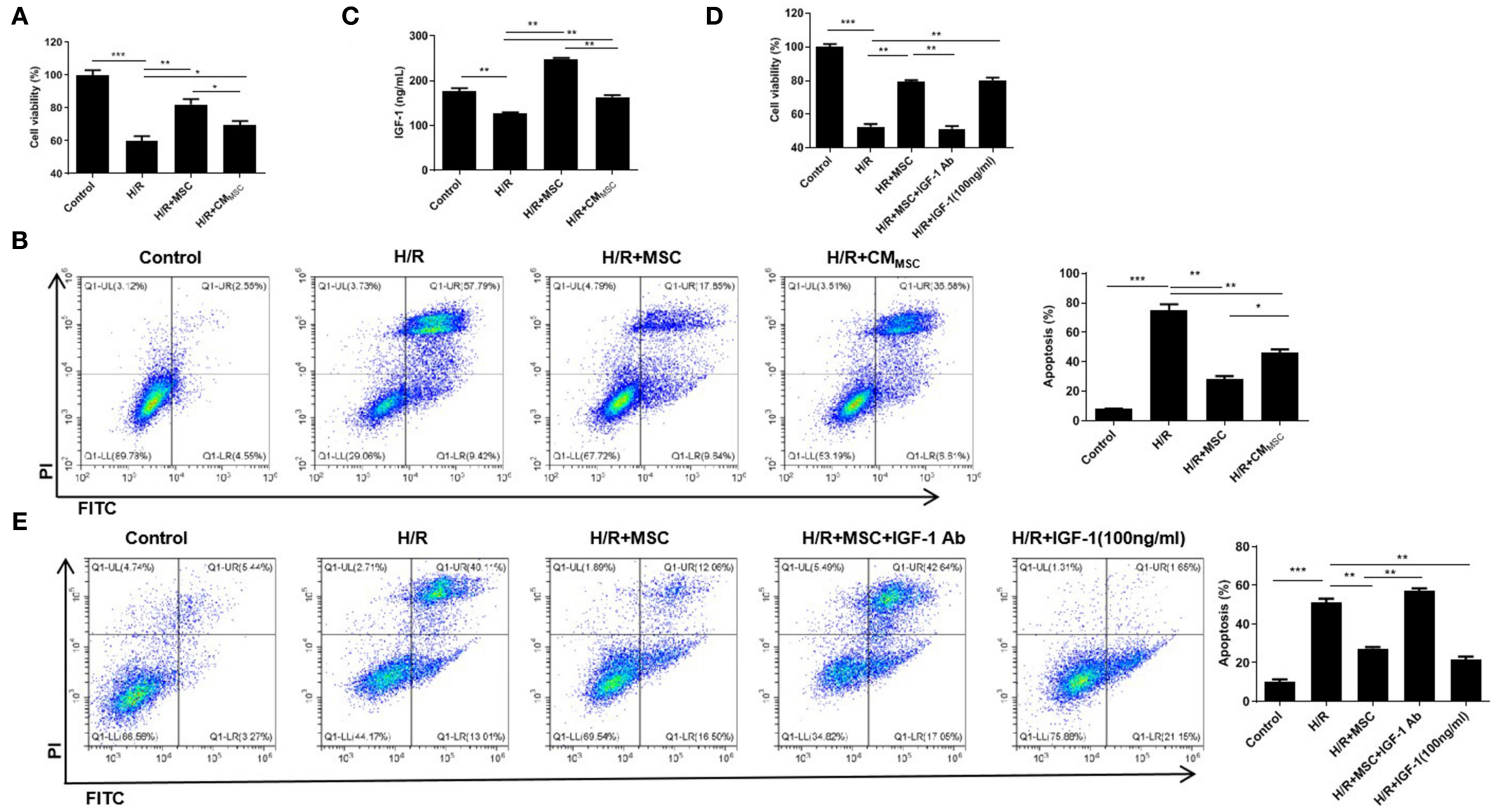
H/R-exposed myocardial cells induce IGF-1 secretion from MSCs to inhibit myocardial injury. **(A)** The viability of H9c2 cells cocultured with MSCs or treated with CM_MSC_ was examined using the CCK-8 assay. **(B)** Cell apoptosis was assessed using flow cytometry. **(C)** ELISA was employed to measure the IGF-1 levels. **(D)** The viability of H9c2 cells cocultured with MSCs and incubated with IGF-1 Ab or IGF-1 was detected using CCK-8 assay. **(E)** Flow cytometry analysis of the apoptosis rate. *P < 0.05, **P < 0.01, and ***P < 0.001.

H9c2 cells were used to establish the H/R model and investigate the involvement of MSCs in myocardial H/R injury. Then, H/R-exposed H9c2 cells were cocultured with MSCs (namely, the H/R+MSC group) or treated with the culture medium of MSCs (namely, the H/R+CM_MSC_ group). The CCK-8 assay revealed that H9c2 cells in the H/R+MSC and H/R+CM_MSC_ groups exhibited a noticeably increased viability compared to the H/R group, and the cell viability of the H/R+MSC group was markedly higher than that of the H/R+CM_MSC_ group (Figure 1A). The flow cytometry results indicated that the H/R group increased the percentage of apoptotic cells, whereas the H/R+MSC and H/R+CM_MSC_ groups exhibited a reduced H/R-mediated apoptosis (Figure 1B). Meanwhile, a markedly higher percentage of apoptotic cells was observed in the H/R+CM_MSC_ group than in the H/R+MSC group (Figure 1B). Next, we investigated the secretion of IGF-1 in the H9c2 cell and MSCs coculture supernatant using an ELISA. Interestingly, the secretion of IGF-1 was increased by the MSC and CM_MSC_ groups compared with the H/R group, and IGF-1 secretion in the H/R+MSC group was higher than that in the H/R+CM_MSC_ group (Figure 1C). Then, H/R-exposed H9c2 cells were cocultured with MSCs and treated with IGF-1 Ab (namely, the H/R+MSC+IGF-1 Ab group). In addition, H/R-exposed H9c2 cells were treated with 100 ng/mL of IGF-1 (namely, the H/R+IGF-1 group). According to the results of the CCK-8 assay, the viability of H/R-exposed H9c2 cells was increased in the H/R+MSC group (Figure 1D). The H/R+MSC+IGF-1 Ab group dramatically reduced the viability of H9c2 cells compared to the H/R+MSC group (Figure 1D). The viability of H/R-exposed H9c2 cells in the H/R+IGF-1 group was also increased, suggesting that IGF-1 promoted H9c2 cell viability (Figure 1D). Similarly, flow cytometry confirmed that IGF-1 inhibited H9c2 cell apoptosis caused by H/R (Figure 1E). Based on the data described above, we concluded that MSCs induced by H/R-exposed H9c2 cells exerted a positive effect on protecting against myocardial injury by secreting IGF-1.

### Exosomes Derived From Damaged Cardiomyocytes Induce MSCs to Secrete IGF-1

**Figure 2:**
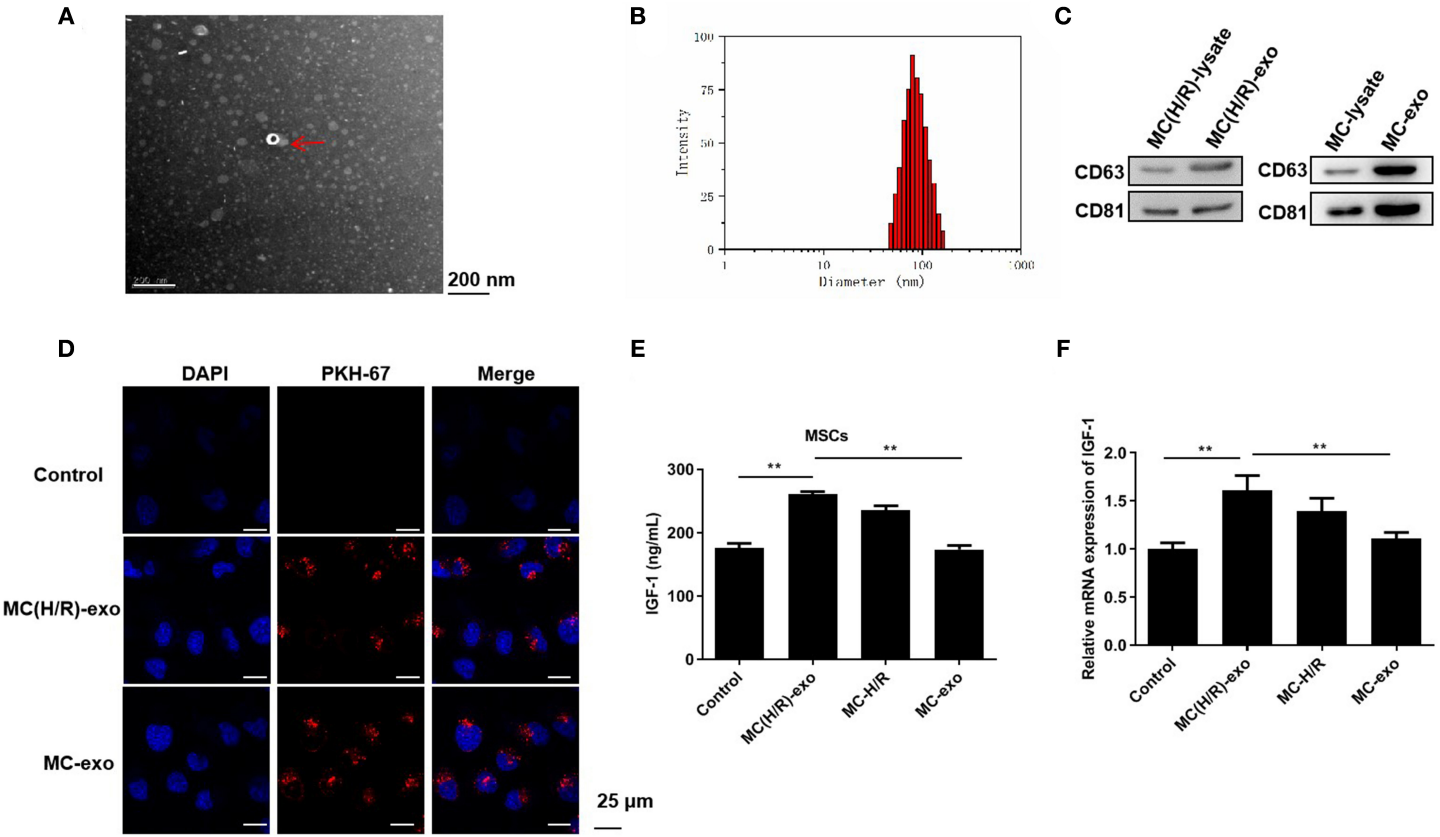
Exosomes derived from damaged cardiomyocytes induce MSCs to secrete IGF-1. **(A)** TEM was performed to scan exosomes isolated from I/R-exposed H9c2 cells. The exosomes were indicated by red arrows (Scale bar = 200 nm). **(B)** NTA was conducted to assess the particle diameter distribution of exosomes. **(C)** Western blotting was applied to verify the expression levels of the exosomal marker proteins CD63 and CD81. **(D)** Immunofluorescence staining was adopted to determine the phagocytosis of PKH-67-labeled exosomes and MSCs (Scale bar = 25 μm). **(E)** ELISA and **(F)** RT-qPCR analysis were employed to assess the IGF-1 levels in MSCs. **P < 0.01.

Subsequently, we elucidated whether H/R-mediated cardiomyocyte-derived exosomes promoted MSCs to secrete IGF-1. Exosomes were initially isolated from the culture supernatant of myocardial cells H9c2 and H/R-exposed H9c2 cells [namely, MC-exo and MC(H/R)-exo]. The extracted particles were characterized using TEM and NTA. These particles were typical round vesicles with diameters of 70–150 nm (Figures 2A,B). Western blot analysis suggested that the particles [MC-exo and MC(H/R)-exo] positively expressed the exosome surface markers CD63 and CD81 (Figure 2C). Collectively, these results confirmed that the extracted particles [MC-exo and MC(H/R)-exo] were exosomes. PKH-67 dye-labeled exosomes were cocultured with MSCs to explore whether the extracted exosomes were absorbed by MSCs. Fluorescence microscopy showed that MC-exo and MC(H/R)-exo were internalized as round vesicles in the cytoplasm of MSCs (Figure 2D). Functionally, IGF-1 secretion in MSCs was dramatically increased by the MC(H/R)-exo treatment (Figure 2E). In addition, MC(H/R)-exo treatment induced the expression of the IGF-1 mRNA (Figure 2F). Therefore, our data implied that exosomes derived from H/R-exposed H9c2 cells induced MSCs to secrete IGF-1.

### The Exosomal lncRNA KLF3-AS1 Mediates the Process by Which Damaged Cardiomyocytes Promote the Secretion of IGF-1 by MSCs

**Figure 3:**
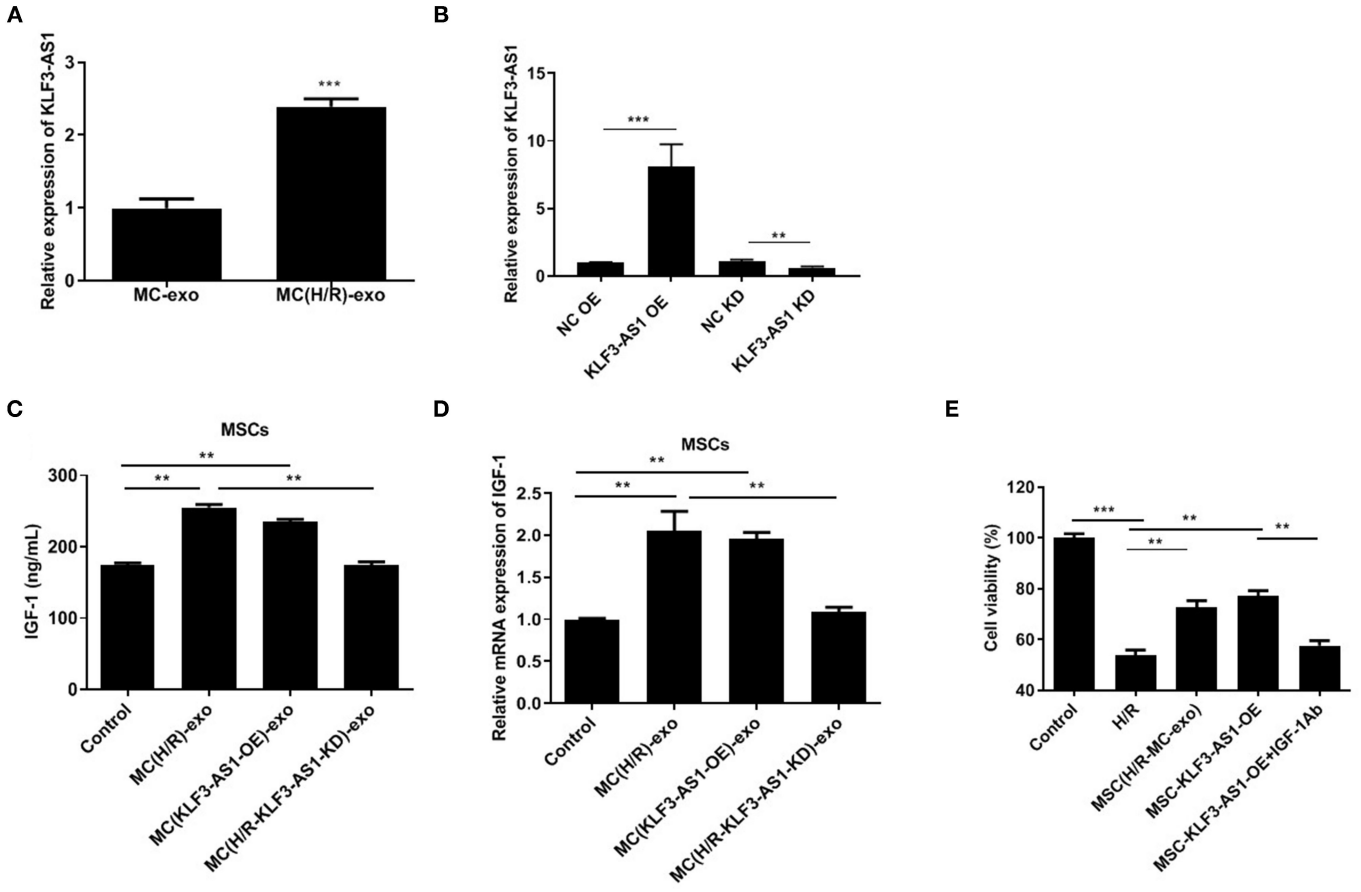
The exosomal lncRNA KLF3-AS1 derived from damaged cardiomyocytes promotes IGF-1 secretion from MSCs. **(A)** The expression level of KLF3-AS1 in MSCs cocultured with exosomes was evaluated using RT-qPCR. **(B)** RT-qPCR was performed to confirm the transfection efficiency of KLF3-AS1. **(C)** ELISA and **(D)** RT-qPCR analysis were employed to assess the IGF-1 levels in MSCs cocultured with exosomes. **(E)** The viability of H9c2 cells treated with exosomes, KLF3-AS1, or IGF-1 Ab was assessed using the CCK-8 assay. **P < 0.01 and ***P < 0.001 compared with the Control group.

RT-qPCR was first performed to assess the KLF3-AS1 expression profile in the MC-exo and MC(H/R)-exo groups and to explore the function of KLF3-AS1 in IGF-1 secretion from MSCs. The expression of KLF3-AS1 in the MC(H/R)-exo group was significantly increased compared with that in the MC-exo group (Figure 3A). Thus, loss-of-function and gain-of-function strategies were employed to explore the biological role of KLF3-AS1. RT-qPCR assays confirmed the efficiency of KLF3-AS1 overexpression and knockdown (Figure 3B). MSCs were treated with exosomes derived from KLF3-AS1-overexpressing H9c2 cells [namely, MC(KLF3-AS1 OE)-exo group]. MSCs were treated with exosomes derived from KLF3-AS1-silenced H/R-exposed H9c2 cells [namely, the MC(H/R-KLF3-AS1-KD)-exo group]. The ELISA and RT-qPCR results revealed that the MC(H/R)-exo group and MC(KLF3-AS1 OE)-exo had significantly increased the IGF-1 levels of MSCs (Figures 3C,D). Moreover, the MC(H/R-KLF3-AS1-KD)-exo group reduced the IGF-1 levels of MSCs compared to the MC(H/R)-exo group (Figures 3C,D). Based on these results, the exosomal lncRNA KLF3-AS1 derived from H/R-exposed H9c2 cells promoted the IGF-1 levels of MSCs. Additionally, MSCs treated with MC(H/R)-exo and overexpression of KLF3-AS1 significantly increased H9c2 cell viability compared to the H/R group, while IGF-1 Ab markedly abrogated the KLF3-AS1-induced increase in cell viability (Figure 3E). Thus, the exosomal lncRNA KLF3-AS1 increased myocardial cell viability by increasing IGF-1 secretion from MSCs.

### The lncRNA KLF3-AS1 Promotes IGF-1 Release by Upregulating STAT5B

**Figure 4:**
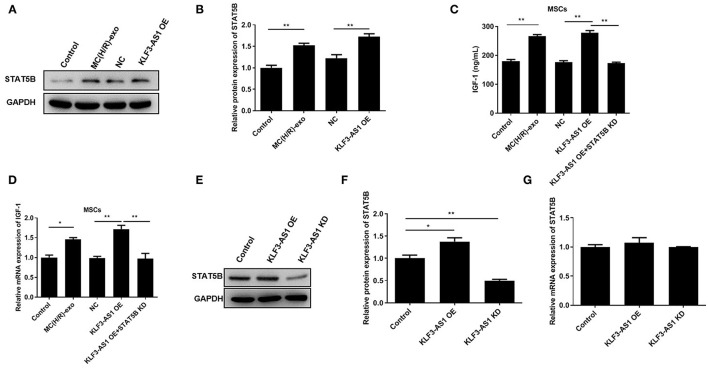
The lncRNA KLF3-AS1 promotes IGF-1 release by upregulating STAT5B. **(A,B)** The expression level of STAT5B in MSCs treated with exosomes or overexpressing KLF3-AS1 was analyzed using Western blotting. **(C)** ELISA and **(D)** RT-qPCR analysis were carried out to detect IGF-1 levels in MSCs treated with exosomes or overexpressing KLF3-AS1. **(E,F)** Western blot and **(G)** RT-qPCR analyses were performed to examine the expression levels of the STAT5B protein and mRNA in MSCs transfected with KLF3-AS1 OE or KLF3-AS1 KD constructs. *P < 0.05 and **P < 0.01.

We explored the regulatory mechanism underlying the involvement of the lncRNA KLF3-AS1 in the occurrence of myocardial injury and predicted STAT5B as a potential target because of its role in regulating IGF-1 (29, 30). Western blots revealed a substantial increase in the expression of the STAT5B protein in the MC(H/R)-exo group and in the overexpressing STAT5B group (Figures 4A,B). In addition, knockdown of STAT5B observably restrained KLF3-AS1-induced increases in IGF-1 levels of MSCs (Figures 4C,D). Subsequently, we discovered that the level of the STAT5B protein was significantly increased in the KLF3-AS1 overexpression group but markedly reduced in the KLF3-AS1 knockdown group compared with the control group, but no significant difference in the STAT5B mRNA level was observed, indicating that the lncRNA KLF3-AS1 positively mediated STAT5B expression through a posttranscriptional regulation (Figures 4E–G). Overall, our data provide evidence that the lncRNA KLF3-AS1 positively modulates STAT5B expression to regulate the IGF-1 levels in MSCs.

### The lncRNA KLF3-AS1 Segregates miR-23c to Promote STAT5B Expression

**Figure 5:**
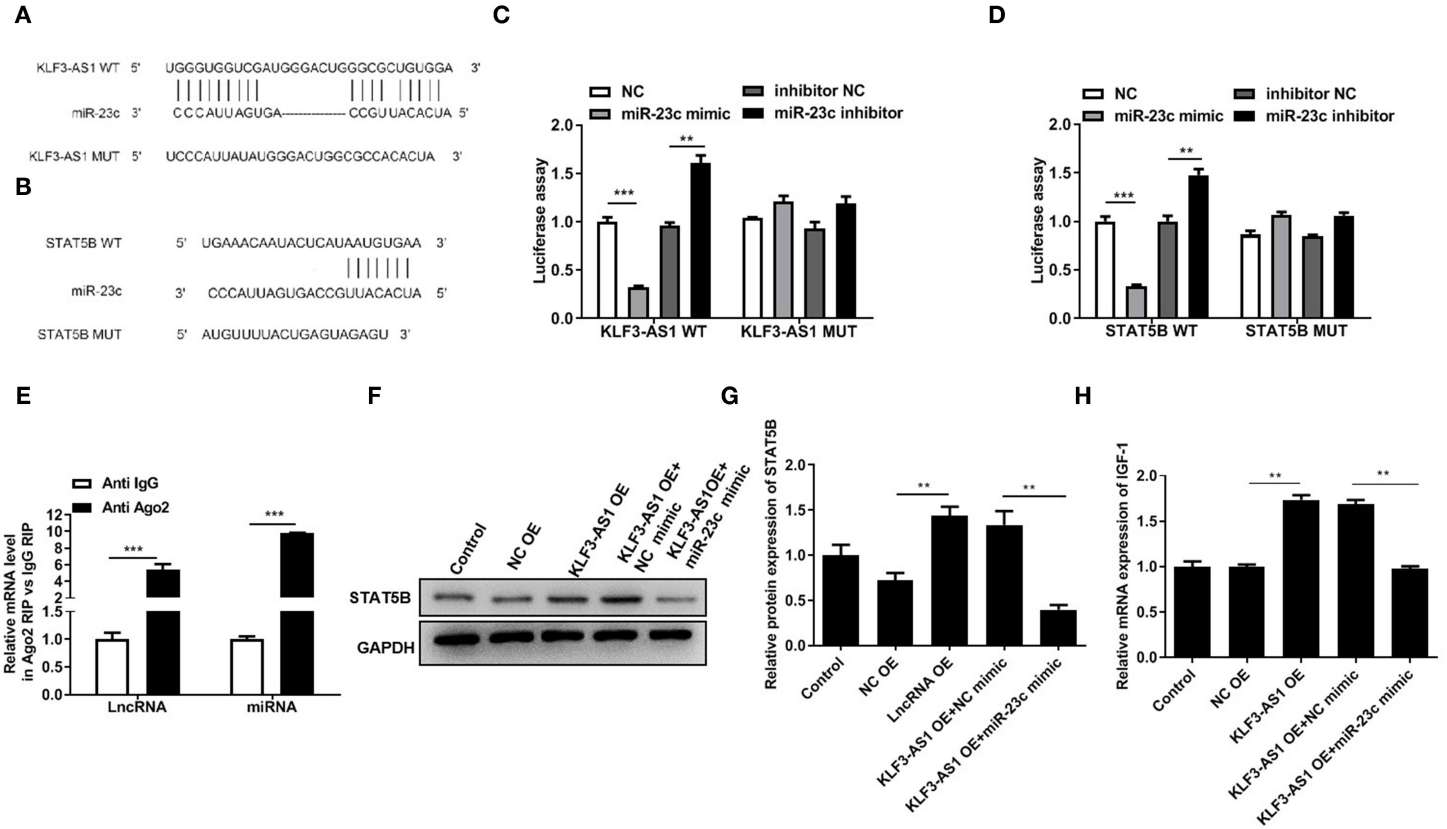
The lncRNA KLF3-AS1 segregates miR-23c to promote STAT5B expression. The putative miR-23c binding sites in the 3′UTR of **(A)** KLF3-AS1 or **(B)** STAT5B. A luciferase reporter assay was employed to analyze the binding relationship between miR-23c and **(C)** KLF3-AS1 or **(D)** STAT5B. **(E)** The binding relationship between KLF3-AS1 and mmiR-23c was validated with a RIP assay. **(F,G)** The expression of the STAT5B protein in MSCs was determined using Western blotting. **(H)** RT-qPCR analysis was used to examine the expression of the STAT5B mRNA in MSCs. **P < 0.01 and ***P < 0.001.

Online bioinformatics databases (StarBase 3.0, TargetScan, and miRDB.org) were used to predict the possible miRNAs mediating the regulatory relationship between lncRNA KLF3-AS1 and STAT5B that participated in modulating the IGF-1 levels. According to bioinformatics databases, miR-23c was chosen for subsequent research because it has potential binding sites for both KLF3-AS1 and STAT5B (Figures 5A,B). Luciferase reporter analyses indicated that the miR-23c mimic significantly reduced luciferase activities in cells cotransfected with KLF3-AS1-WT or STAT5B-WT, while the luciferase activity of KLF3-AS1-WT or STAT5B-WT was notably increased by the miR-23c inhibitor (Figures 5C,D). However, miR-23c did not produce a noticeable difference in the luciferase activity of KLF3-AS1-Mut and STAT5B-Mut (Figures 5C,D). In addition, KLF3-AS1 and miR-23c were more abundant in the anti-Ago2-treated immunoprecipitates than in the anti-IgG-treated immunoprecipitates (Figure 5E). Meanwhile, we verified that the miR-23c mimic partially abrogated the increased expression of the STAT5B protein caused by the overexpression of KLF3-AS1, as evidenced by Western blotting (Figures 5F,G). Functionally, forced expression of KLF3-AS1 increased the expression of the IGF-1 mRNA, and the miR-23c mimic counteracted the effect of KLF3-AS1 on IGF-1 expression (Figure 5H). Based on these results, the lncRNA KLF3-AS1 increased the IGF-1 levels by regulating miR-23c/STAT5B.

### The Exosomal lncRNA KLF3-AS1 Induces IGF-1 Secretion From MSCs to Inhibit Myocardial Injury *in vivo*

**Figure 6:**
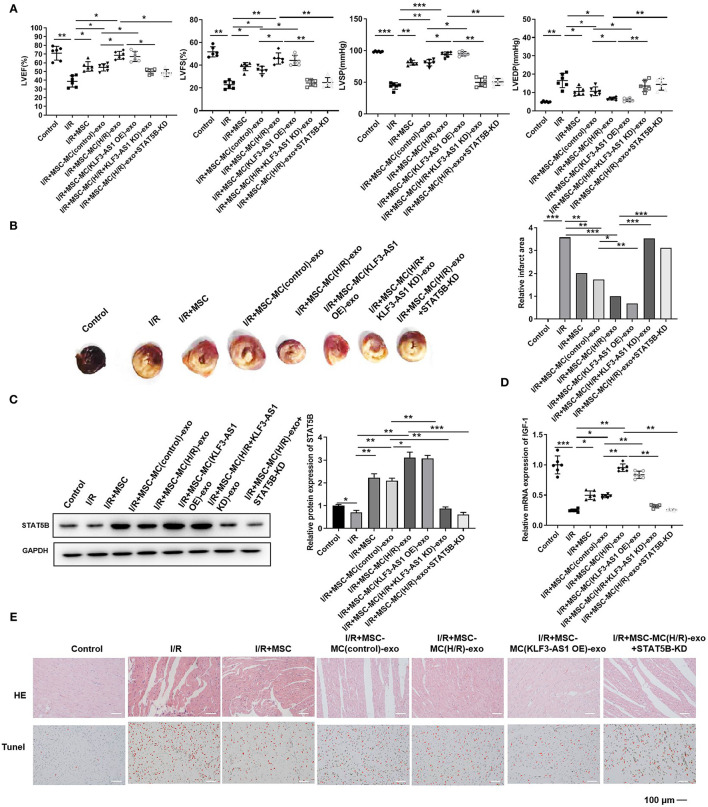
The exosomal lncRNA KLF3-AS1 induces IGF-1 secretion from MSCs to inhibit myocardial injury *in vivo*. **(A)** Echocardiography was used to assess the cardiac function, such as LVEF, LVFS, LVSP, and LVEDP. **(B)** The infarct area of I/R-injured rats was detected by performing TTC staining. **(C)** The expression of STAT5B in I/R-injured rats was measured using Western blotting. **(D)** RT-qPCR analysis was implemented to measure the expression level of the IGF-1 mRNA in I/R-injured rats. **(E)** HE staining and TUNEL assays were performed to estimate the degree of myocardial tissue damage. The apoptosis cells were indicated by red arrows (Scale bar = 100 μm). For the TUNEL assay, apoptotic cells are indicated by red arrows. *P < 0.05, **P < 0.01, and ***P < 0.001.

Subsequently, we confirmed the effects of the exosomal lncRNA KLF3-AS1 on myocardial injury *in vivo*. We established a myocardial I/R model in rats and injected MSCs exposed to different treatments into the myocardium; then, echocardiography was used to assess the cardiac function. LVEF, LVFS, and LVSP were significantly reduced in the I/R group, while LVEDP was increased in the I/R group (Figure 6A). The I/R+MSC, I/R+MSC-MC(control)-exo, and I/R+MSC-MC(H/R)-exo groups exhibited significantly increased LVEF, LVFS, and LVSP and decreased LVEDP compared with the I/R group (Figure 6A). The I/R+MSC-MC(control)-exo group displayed higher LVEF, LVFS, and LVSP and a lower LVEDP than the I/R+MSC-MC(H/R)-exo group (Figure 6A). The I/R+MSC-MC(KLF3-AS1 OE)-exo group had increased LVEF, LVFS, and LVSP and a decreased LVEDP compared with the I/R+MSC-MC(control)-exo group (Figure 6A). The I/R+MSC-MC(H/R+KLF3-AS1 KD)-exo and I/R+MSC-MC(H/R)-exo+STAT5B-KD groups exhibited decreased LVEF, LVFS, and LVSP and an increased LVEDP compared with the I/R+MSC-MC(H/R)-exo group (Figure 6A). TTC staining showed a larger infarct area in the I/R group than in the control group (Figure 6B). The I/R+MSC, I/R+MSC-MC(control)-exo, and I/R+MSC-MC(H/R)-exo groups displayed significantly decreased infarct areas compared with the I/R group, and the infarct size of the I/R+MSC-MC(control)-exo group was significantly larger than that of the I/R+MSC-MC(H/R)-exo group (Figure 6B). The I/R+MSC-MC(KLF3-AS1 OE)-exo group presented a decreased infarct area compared with the I/R+MSC-MC(control)-exo group. The infarct area was increased in the I/R+MSC-MC(H/R+KLF3-AS1 KD)-exo and I/R+MSC-MC(H/R)-exo+STAT5B-KD groups compared with the I/R+MSC-MC(H/R)-exo group. The Western blot results showed substantial increases in the levels of the STAT5B protein in the I/R+MSC-MC(H/R)-exo and I/R+MSC-MC(KLF3-AS1 OE)-exo groups compared with the I/R+MSC-MC(control)-exo group (Figure 6C). In addition, the STAT5B level was reduced in the I/R+MSC-MC(H/R+KLF3-AS1 KD)-exo and I/R+MSC-MC(H/R)-exo+STAT5B-KD groups compared with the I/R+MSC-MC(H/R)-exo group (Figure 6C). Similarly, the I/R+MSC-MC(H/R)-exo and I/R+MSC-MC(KLF3-AS1 OE)-exo groups exhibited noticeably increased IGF-1 levels compared with the I/R+MSC-MC(control)-exo group (Figure 6D). In addition, the I/R+MSC-MC(H/R+KLF3-AS1 KD)-exo and I/R+MSC-MC(H/R)-exo+STAT5B-KD groups exhibited reduced IGF-1 levels compared with the I/R+MSC-MC(H/R)-exo group (Figure 6D). Moreover, H&E staining and TUNEL assays showed that the I/R+MSC-MC(H/R)-exo and I/R+MSC-MC(KLF3-AS1 OE)-exo groups displayed noticeably decreased abnormal pathological alterations and apoptosis in rat myocardial tissues compared with the I/R+MSC-MC(control)-exo group (Figure 6E). The I/R+MSC-MC(H/R+KLF3-AS1 KD)-exo and I/R+MSC-MC(H/R)-exo+STAT5B-KD groups exhibited increased abnormal pathological alterations and apoptosis in rat myocardial tissues compared with the I/R+MSC-MC(H/R)-exo group (Figure 6E). Taken together, the lncRNA KLF3-AS1 dramatically abrogated the apoptosis of rat myocardial tissues by regulating IGF-1 secretion *in vivo*.

## Discussion

Myocardial I/R injury is considered an inevitable pathological process after myocardial infarction revascularization that causes myocardial microcirculation disorder and myocardial cell apoptosis (31). A cardioprotective measure to prevent and treat ischemia-reperfusion injury and minimize the apoptosis rate of myocardial cells must be developed to improve the prognosis of patients with myocardial infarction. These findings suggested a protective role for IGF-1 secreted from MSCs in myocardial I/R injury.

MSCs represent a novel therapeutic method because they modulate many regenerative and immunomodulatory processes; however, the underlying biological mechanism of MSC-mediated repair remains unclear (32). MSCs have been reported to be attractive candidates for the inflammatory modulation of myocardial I/R injury (33). In addition, MSCs regulate macrophage polarization to exert a therapeutic effect on myocardial I/R injury (34). Some studies have suggested that MSCs exert their effects through developmental plasticity (35). Here, we proved that growth factors exerted an effective therapeutic effect on tissue injury by activating paracrine signaling mechanisms (36, 37). One of these growth factors is IGF-1, an extracellular polypeptide signaling molecule (38). In recent years, the important role of IGF-1 in the occurrence and development of cardiovascular diseases has attracted increasing attention (39). Boucher et al. found that IGF-1 and darbepoetin Alfa effectively promote angiogenesis and reduce myocardial infarction (40). In addition, Sliva et al. documented that MSCs transfected with the IGF-1 cDNA remarkably inhibit cardiac inflammation and fibrosis (41). In the current study, H/R-exposed H9c2 cells induced MSCs to promote cell viability and inhibit cell injury by secreting IGF-1.

Paracrine signaling plays a vital role in improving the local microenvironment of myocardial infarction and repairing the infarcted myocardium; among which, exosomes are the main substances involved in paracrine pathways (42, 43). For instance, exosomes derived from cardiac fibroblasts protect cardiomyocytes from H/R injury (44). Additionally, exosomes from epigallocatechin gallate-treated cardiomyocytes attenuate apoptosis and autophagy in cardiomyocytes (45). As shown in our present study, exosomes derived from the damaged myocardium induced MSCs to secrete IGF-1, suggesting a role in the regulation of I/R injury.

Exosomes mediate intercellular communication and macromolecule transfer of biologically active substances, and exosomal lncRNAs have attracted immense research interest worldwide because of their better conservation and stability (46, 47). Hence, dissecting the role of exosomal lncRNAs in I/R injury progression is important for diagnosis and clinical treatment. LncRNAs recruit chromatin-modified complexes for transcriptional regulation and interact with miRNAs, mRNAs, and/or proteins for posttranscriptional regulation (48). The lncRNA KLF3-AS1 was expressed at high levels in exosomes derived from H/R-treated H9c2 cells. Moderate evidence suggesting the positive effects of the lncRNA KLF3-AS1 on the biological processes involved in many diseases is available. For instance, the lncRNA KLF3-AS1 functions as a tumor suppressor by targeting miR-185-5p/KLF3 in esophageal squamous cell carcinoma (25). According to a recent study, KLF3-AS1 promotes cartilage repair and chondrocyte proliferation by impairing the miR-206-mediated inhibition of GIT1 in osteoarthritis (49). Hypoxia-induced exosomes from H9c2 cells were loaded with heart-protective active substances that alleviate cell apoptosis (50). In the present study, we provided a direct evidence that exosomal KLF3-AS1 inhibited myocardial injury by promoting IGF-1 secretion from MSCs. In addition, bioinformatics analysis and luciferase reporter assays proved that KLF3-AS1 sponged miR-23c to induce STAT5B expression. Recently, several studies have reported that STAT5B induces IGF-1 expression in response to different physiological processes (51, 52). Sheng et al. reported that the JAK-STAT-IGF-1 pathway inhibited cell apoptosis in H/R-exposed H9c2 cells (53). In addition, we further investigated whether the lncRNA KLF3-AS1 upregulated the level of IGF-1 to reduce cell apoptosis by regulating miR-23c/STAT5B signaling.

Overall, these findings indicated that damaged myocardium-derived exosomal KLF3-AS1 promoted IGF-1 secretion from MSCs to reduce myocardial I/R injury by regulating the miR-23c/STAT5B axis. In summary, our study provides insights into a novel target for the treatment of myocardial I/R injury.

## Data Availability Statement

The raw data supporting the conclusions of this article will be made available by the authors, without undue reservation.

## Ethics Statement

The animal study was reviewed and approved by the Institutional Animal Care and Use Committee of Taizhou People's Hospital.

## Author Contributions

GC and AY conceived the study, wrote the manuscript, and designed the figures. MW, ZR, and LZ contributed to the writing and editing of the manuscript. All authors contributed to the article and approved the submitted version.

## Funding

This work was supported by the Jiangsu Young Medical Key Talents Fund Project (Project No. QNRC2016510) and the General Project of Jiangsu Health Committee (Project No. H2018003).

## Conflict of Interest

AY was employed by company Taizhou Mabtech Pharmaceutical Co., Ltd. The remaining authors declare that the research was conducted in the absence of any commercial or financial relationships that could be construed as a potential conflict of interest.

## Publisher's Note

All claims expressed in this article are solely those of the authors and do not necessarily represent those of their affiliated organizations, or those of the publisher, the editors and the reviewers. Any product that may be evaluated in this article, or claim that may be made by its manufacturer, is not guaranteed or endorsed by the publisher.

## Abbreviations

I/R: ischemia-reperfusion
MSCs: mesenchymal stem cells
IGF-1: insulin-like growth factor 1
H/R: hypoxia-reoxygenation
lncRNAs: long noncoding RNAs
miRNAs: microRNAs
mRNAs: messenger RNAs
NC: negative control
CCK-8: Cell Counting Kit-8
TEM: transmission electron microscopy
NTA: nanoparticle tracking analysis
WT: wild-type
MUT: mutant
RIP: RNA immunoprecipitation
H&E: hematoxylin and eosin
SD: standard deviation.
